# Searching for longevity hotspots in Denmark

**DOI:** 10.18632/aging.101579

**Published:** 2018-10-13

**Authors:** Anne Vinkel Hansen, Laust Hvas Mortensen, Rudi Westendorp

**Affiliations:** 1 Statistics Denmark, Copenhagen, Denmark; 2 Department of Public Health, University of Copenhagen, Copenhagen, Denmark; 3 Center for Healthy Aging, University of Copenhagen, Copenhagen, Denmark

**Keywords:** centenarian rate, longevity, Denmark, cluster detection

## Abstract

While existing research on regions with high prevalence of centenarians has focused on selected candidate geographical regions, we explore the existence of hotspots in the whole of Denmark.

We performed a Kulldorff spatial scan, searching for regions of birth, and of residence at age 71, where an increased percentage of the cohort born 1906-1915 became centenarians. We then compared mortality hazards for these regions to the rest of the country.

We found a birth hotspot of 222 centenarians, 1.37 times more than expected, centered on a group of rural islands. Lower mortality hazards from age 71 onwards were confined to those born within the hotspot and persisted over a period of at least 30 years. At age 71, we found two residence-based hotspots of 348 respectively 238 centenarians, 1.46 and 1.44 times the expected numbers. One hotspot, located in high-income suburbs of the Danish capital, seems driven by selective in-migration of low-mortality individuals. The other hotspot seems driven by selective migration and lower morality among those born and residing in the hotspot.

Thus, Danish centenarian hotspots do exist. The locations and interpretation depend on whether we look at place of birth or of residence late in life.

## Introduction

Those who reach hundred years of age capture our imagination. While the specific number is an arbitrary marker, becoming a centenarian is not a meaningless indicator of longevity, and the interest in geographical regions where a high proportion of people become centenarians has been ongoing at least since the start of the 20th century [1]. The first of the current generation of well-validated longevity regions is the Sardinian “blue zone” [2], a small group of villages where a particularly large percentage of those born there from 1880 to 1900 become centenarians. Other “blue zones” have been identified in the Okinawa region in Japan [3], the Ikaria Island in Greece [4] and the Nicoya peninsula in Costa Rica [5].

A plethora of explanatory factors has been proposed, roughly separating into seeing any effect as either genetic, rooted in local culture, or caused by local physical environment. For high-longevity zones in Calabria, Italy, Montesanto et al. [6] note a low variety of surnames, hinting at an explanation grounded in inbreeding. Topographic factors, particularly altitude and steepness of terrain have been suggested [7]. Life style factors have been investigated with varying results - the population of the Nicoya region has a diet rich in fibers, proteins and trans fats [5], while Okinawa is the only blue zone to have a significantly lower caloric intake than the reference population [8]. For the Nicoya blue zone, it has been observed that the effect is exclusive to those born and resident in the Nicoya region, with a non-significant decrease in mortality among immigrants and no decrease in mortality among out-migrants [5]. This hints at the effect being, in some way, one of physical environment, or at least not tied to cultural factors carried along by out-migrants.

So far, the known longevity hotspots are in isolated, economically disadvantaged regions. In addition, the known longevity hotspots have been identified by spatial smoothing methods (as detailed in [2]) after first selecting a candidate region based on available statistics. This paper takes a different approach. What if longevity hotspots arise at random when conditions align? If this is the case, then it is possible that hotspots can be identified by scanning across seemingly homogeneous regions without any known candidate regions. As places change over time (sanitation improves, public transportation replaces walking, pesticide use changes), exposure to a place may mean different things in different decades. To our knowledge, there has not been any exploration of whether the effects observed in the various blue zones persist in generations after the ones they were detected in.

In terms of longevity, Denmark is a curious case. The country is small, socially homogeneous, highly economically developed, and has one of the world's most generous universal welfare systems. At the same time, Denmark also has considerable social inequalities in health and a comparatively modest life expectancy of 80.7 years in 2016, compared to 80.6 for the EU in general and 78.7 for the US [9]. This study explores whether centenarian hotspots based on place of birth and place of residence late in life exist in Denmark. We explore the effects of time by examining whether such hotspots also prolong life in those moving there at a later age, and whether the effects persist in subsequent generations.

## RESULTS

There were 740,927 live births in Denmark from 1906 to 1915 [10]. Of these, we found 425,791 still alive and resident in Denmark by age 71. In order to make analyses by geographic location of birth, we made exclusions as follows: Those emigrating between age 71 and 100 (n = 1107), those who had no records past a certain year and also no record of death or emigration (n = 829), those with no parish of birth recorded (n = 57,195) and those missing information on parish of residence by age 71 (n=4,596). Of the group missing parish of birth, the majority (n = 42,436) had a record of municipality of birth in place of parish. Of the 362,064 individuals remaining in the study, 4,739 (1.3%) reached the age of 100, and 520 (0.1%) survived past the end of the observation period (Dec 31^st^ 2015). Of the group excluded, 1.3% became centenarians – although the proportion was 1.6 for those with municipality but not parish of birth recorded.

### Hotspots by place of birth

**Figure 1:**
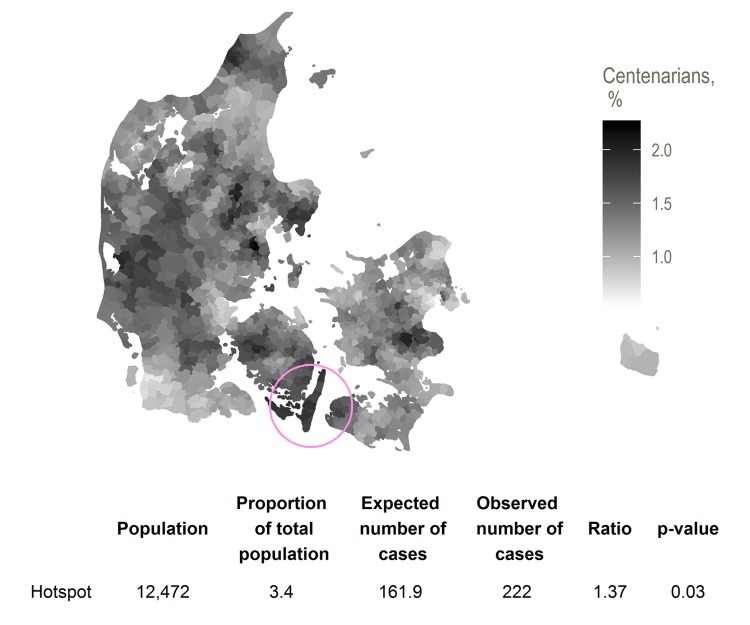
Hotspot for proportion of birth cohort surviving from 71 to 100, with smoothed centenarian proportions.

When scanning for hotspots by place of birth, we found one hotspot centered on a group of rural islands (Langeland and encompassing rural areas on the islands of Funen and Lolland – see Figure 1). The birth hotspot had 222 centenarians, 1.37 times the expected number, and the proportion was significantly larger than that of the remaining country with a p-value of 0.03. As shown in Table 1, those born in the birth hotspot did not differ markedly from the baseline population on socioeconomic factors, except in being more likely to be homeowners (67% vs 59%).

**Table 1:** Socio-economic characteristics at age 71 for the study population and the birth-cohort centenarian hotspot .

		**Study population**			**Birth-cohort hotspot**	
		**N**	**%**		**N**	**%**
**Sex**						
	Female	198,034	54.7		6,773	54.3
	Male	164,030	45.3		5,699	45.7
**Socioeconomic position in 1970**						
	1 (highest)	14,169	3.9		476	3.8
	2	22,739	6.3		763	6.1
	3	93,278	25.8		3,164	25.4
	4	65,195	18		2,035	16.3
	5 (lowest)	89,038	24.6		3,285	26.3
	Pensioner	70,348	19.4		2,471	19.8
	Other/unknown	7,297	2		278	2.2
**Marital status in 1970**						
	Divorced	18,750	5.2		547	4.4
	Married	270,997	74.8		9,377	75.2
	Unmarried	31,898	8.8		1,175	9.4
	Widow(er)	38,370	10.6		1,312	10.5
	Unknown	2,049	0.6		61	0.5
**Homeowner in 1970**						
	No	147,632	40.8		4,156	33.3
	Yes	214,432	59.2		8,316	66.7

Mortality differences between those born inside and outside the birth hotspot were more pronounced for women (HR 0.93, 95% CI 0.91 – 0.95) than for men (HR 0.97, 95% CI 0.95 – 1.00). This corresponds to the proportion surviving from 71 to 100 being 2.8% and 1.9% inside and outside the birth hotspot for women, and 0.6% and 0.5% for men (Table 2).

**Table 2:** Mortality and centenarian proportion for those born in and outside the birth cohort centenarian hotspot, by sex and by place of residence at age 71. HR computed by Cox regression.

		**N**	**Alive by age 100, ** **N (%)**		**HR (95% CI)**	Adjusted* HR (95% CI)
				
**Hotspot**						
	Born outside hotspot	349,592	4,517 (1.3)	1 (REF)		1 (REF)
	Born in hotspot	12,472	222 (1.8)	0.95 (0.93 – 0.97)		0.95 (0.94 – 0.97)
				
**Sex**						
	Men born outside hotspot	158,331	793 (0.5)	1 (REF)		1 (REF)
	Men born in hotspot	5,699	35 (0.6)	0.97 (0.95 – 1.00)		0.98 (0.96 1.01)
					
	Women born outside hotspot	191,261	3,724 (1.9)	1 (REF)		1 (REF)
	Women born in hotspot	6,773	187 (2.8)	0.93 (0.91 – 0.95)		0.94 (0.91 – 0.96)
					
**Place of birth and residence at age 71**						
	Born and stayed outside hotspot	346,230	4,473 (1.3)	1 (REF)		1 (REF)
	Born outside and moved to hotspot	3,362	44 (1.3)	0.99 (0.96 – 1.03)		1.01 (0.97 – 1.04)
	Born in and left hotspot	6,652	120 (1.8)	0.96 (0.94 – 0.98)		0.96 (0.93 – 0.98)
	Born and stayed in hotspot	5,820	102 (1.8)	0.94 (0.92 – 0.97)		0.95 (0.92 – 0.97)
					

*Adjusted for birth year, socioeconomic position, marital status and homeownership.

Mortality for those born in the birth hotspot and still remaining there by age 71 was slightly lower than for those who had left the birth hotspot (HR when compared to those born and remaining outside the hotspot 0.94, 95% CI 0.92 - 0.97 and 0.96, 95% CI 0.94 – 0.98 respectively) but with overlapping confidence intervals. There was no decrease in mortality for those born outside the birth hotspot but resident in the birth hotspot by age 71 (HR 0.99, 95% CI 0.96 – 1.03). The centenarian proportions reflect this pattern: the proportion surviving to 100 is 1.3% for those born outside and 1.8% for those born inside the birth hotspot, wherever they live by age 71. Adjustment for birth year, socioeconomic position, marital status and homeownership did not materially change the effect sizes.

Age-specific mortality rates for the cohorts born in the birth hotspot in the periods 1916-25 and 1926-35 are shown in the Table 3. Age-specific mortality rates were comparable across birth cohorts, and remain significantly lower than expected from age 86 and onwards.

**Table 3:** Socio-economic characteristics at age 71 for the study population and the residence-based centenarian hotspots.

		**Study population**			**Primary residence-based hotspot**			**Secondary residence-based hotspot**	
		**N**	**%**		**N**	**%**		**N**	**%**
**Sex**									
	Female	198,034	54.7		10,096	57.1		6,725	51.2
	Male	164,030	45.3		7,580	42.9		6,408	48.8
**Socioeconomic position in 1970**									
	1 (highest)	14,169	3.9		2,157	12.2		469	3.6
	2	22,739	6.3		2,422	13.7		751	5.7
	3	93,278	25.8		4,870	27.6		3,798	28.9
	4	65,195	18.0		3,062	17.3		2,063	15.7
	5 (lowest)	89,038	24.6		2,433	13.8		3,376	25.7
	Pensioner	70,348	19.4		2,311	13.1		2,441	18.6
	Other/unknown	7,297	2		421	2.4		235	1.8
**Marital status in 1970**									
	Divorced	18,750	5.2		1,374	7.8		305	2.3
	Married	270,997	74.8		12,999	73.5		10,323	78.6
	Unmarried	31,898	8.8		1,403	7.9		1,282	9.8
	Widow(er)	38,370	10.6		1,794	10.1		1,160	8.8
	Unknown	2,049	0.6		106	0.6		63	0.5
**Homeowner in 1970**									
	No	147,632	40.8		9,186	52		2,483	18.9
	Yes	214,432	59.2		8,490	48		10,650	81.1

A sensitivity analysis where the group coded with municipality rather than parish of birth was not excluded (for a total n=404,500) found the same primary hotspot, with 240 centenarian cases, 1.33 times the expected number with a p-value of 0.10.

### Hotspots by place of residence at age 71

Here, we found one primary hotspot with 1.46 times (p-value 0.001), and one secondary hotspot with 1.44 times (p-value 0.001) the expected number of centenarians. The primary residence hotspot consisted of parishes in generally high-income suburbs in Northern Zealand. The secondary residence hotspot covered a region in Mid-Jutland. The total number of centenarians in each region was 348 and 238, respectively, for the primary and secondary residence hotspots. There were no further secondary clusters.

The primary residence hotspot in Northern Zealand showed a higher proportion with a high socioeconomic position (26% in the two highest groups, compared to 10% for the cohort in general) and a higher proportion of divorcees (Table 3) whereas no such differences were observed for the secondary residence hotspot in Mid-Jutland.

Table 4 presents the matching mortality hazards for the residence-based hotspot from age 71 onwards. Within the primary hotspot, the lower mortality hazards were confined to those who moved into the hotspot (HR 0.88, 95% CI 0.87 – 0.90). Those born in the hotspot did not show any benefit, whether staying in or leaving the region (1.00, 95% CI 0.96 – 1.04 and 1.00, 95% CI 0.96 – 1.05 respectively). In the second residence-based hotspot, those who moved into the hotspot showed significant lower mortality hazards (0.96, 95% CI 0.93 – 0.98) which is comparable to those who were born and stayed in the region (0.95, 95% CI 0.92 – 0.97).

**Table 4:** Mortality and centenarian proportion for those born in and outside the residence-based centenarian hotspots, by sex and by place of residence at age 71. HR computed by Cox regression.

		**N**	**Alive by age 100, ** **N (%)**	**HR (95% CI)**	Adjusted* HR (95% CI)
				
**Hotspots**					
	Resident outside hotspots	331,255	4,153 (1.3)	1 (REF)	1 (REF)
	Primary hotspot	17,676	348 (2.0)	0.89 (0.88 – 0.91)	0.91 (0.89 – 0.93)
	Secondary hotspot	13,133	238 (1.8)	0.95 (0.93 – 0.96)	0.96 (0.95 – 0.98)
				
**Sex**					
	Men resident outside hotspots	150,042	714 (0.5)	1 (REF)	1 (REF)
	Men in primary hotspot	7,580	58 (0.8)	0.91 (0.89 – 0.93)	0.94 (0.92 – 0.97)
	Men in secondary hotspot	6,408	56 (0.9)	0.92 (0.90 – 0.94)	0.94 (0.92 – 0.96)
					
	Women resident outside hotspots	181,213	3,439 (1.9)	1 (REF)	1 (REF)
	Women in primary hotspot	10,096	290 (2.9)	0.90 (0.88 – 0.92)	0.92 (0.90 0.94)
	Women in secondary hotspot	6,725	182 (2.7)	0.94 (0.92 – 0.97)	0.96 (0.94 – 0.99)
				
**Movement out of and into primary hotspot**					
	Born and stayed outside hotspot	341,707	4,361 (1.3)	1 (REF)	1 (REF)
	Born outside and moved to hotspot	15,610	317 (2.0)	0.88 (0.87 – 0.90)	0.90 (0.89 – 0.92)
	Born in and left hotspot	2,681	30 (1.1)	1.00 (0.96 – 1.04)	1.00 (0.96 – 1.03)
	Born and stayed in hotspot	2,066	31 (1.5)	1.00 (0.96 – 1.05)	1.00 (0.96 – 1.05)
				
**Movement out of and into secondary hotspot**					
	Born and stayed outside hotspot	340,103	4,374 (1.3)	1 (REF)	1 (REF)
	Born outside and moved to hotspot	5,473	108 (2.0)	0.96 (0.93 – 0.98)	0.97 (0.95 – 1.00)
	Born in and left hotspot	8,828	127 (1.4)	0.95 (0.92 – 0.96)	0.95 (0.93 – 0.97)
	Born and stayed in hotspot	7,660	130 (1.7)	0.95 (0.92 – 0.97)	0.96 (0.94 – 0.98)

*Adjusted for birth year, socioeconomic position, marital status and homeownership.

**Figure 2:**
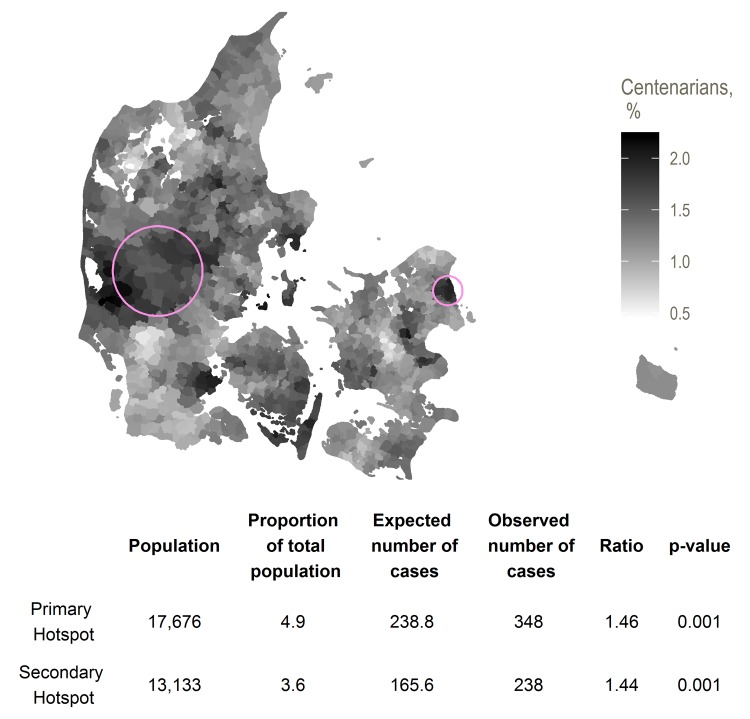
Primary (eastmost) and secondary (westmost) hotspots for proportion of residents in a region at age 71 surviving to age 100, with smoothed centenarian proportions

Of the 12,472 subjects born in the birth hotspot, 453 (3.6%) and 59 (0.5%) were living in the primary and secondary residence hotspots by age 71, compared to 4.9% and 3.6% for the total population (Figure 2). A Cox regression including indicators for being born in the birth hotspot and for living in each of the residence hotspots at age 71 showed no interaction between the birth and residence hotspots (p-value 0.92, data not shown).

## DISCUSSION

We discovered a Danish longevity birth hotspot centered on a group of rural islands, with a 1.37 times increased chance of becoming a centenarian for the cohort born 1906-1915. The hotspot has lower post-71 mortality for both men and women, although markedly more so for women. Mortality is lower for all those born in the hotspot, whether or not they are still living there by age 71. The difference in mortality is still observable and not substantially weakened for women born in the hotspot 1916-25 and 1926-35. We find two regions with significantly increased probabilities of reaching age 100 for those resident there at age 71, both with centenarian rates similar to those of the birth hotspot.

The main strength of this study is that the use of routinely collected, nation-wide registry data allows us to explore longevity distribution across an entire country for a 10-year birth cohort, that we have full follow-up from at least the age of 71, and that we can examine the effect in subsequent birth cohorts. One weakness is that we only have follow-up from 1977 and thus are unable to examine the extent to which our effects are shaped by mortality or emigration earlier in life. We only measure place of residence at two points in life, although people may have moved in and out of the regions studied. We do not have information on length of life after emigration, which in case of geographically skewed migration might bias results.

The Kulldorff cluster detection method is known to be conservative [11], and is constructed for point data, not area-aggregated data as we used. A likely effect of aggregation is over-estimation of cluster sizes [12], which, in turn, would reduce the estimate size. The method allows for hotspots that group together regions separated by sea – this may seem counterintuitive, but arguably, a short stretch of sea is not necessarily a boundary in terms of local culture, population mixing or socioeconomic factors.

Although nearly 10% of observations were excluded due to missing information on parish of birth, sensitivity analyses including these observations did not change the location of the birthplace hotspot, and while the effect size decreased past the point of statistical insignificance, the absolute change was only from a ratio of 1.37 to a ratio of 1.33. As noted above, we would expect effect sizes to decrease when including data aggregated to larger geographical units.

The absolute centenarian proportions in the study are not large. Comparison between studies are complicated by different outcome measures and especially different study periods, but the relative increase in probability of becoming a centenarian of 37% can be compared to the 50% increase in probability for the Sardinian blue zone or the three-fold increase in probability for the Sardinian restricted blue zone [1]. Similarly, the age 70-100 death rate ratio of 0.95 for the Langeland hotspot compares to the death rate ratio of 0.8 for Nicoyan men [5]. Given the relatively high geographic, economic and cultural homogeneity in Denmark, it is not surprising that relative differences in centenarian proportions within Denmark are moderate.

Whatever the causes of the observed increases in extreme longevity for those born in birth hotspot, they seem to be determined before age 71. We see comparable post-71 mortality estimates for those born and staying in and leaving the hotspot, and no decrease in mortality for those moving to the birth hotspot as compared to those born and remaining outside the hotspot. The fact that there seems to be no benefits to moving to the birth hotspot is reinforced by the results of the scan for residence-based hotspots – the best place to be born is not the best place to grow old. This could point to the causes of the increase in centenarian prevalence being genetic or related to early life exposures, or being rooted in behaviors that are learned before old age and continued after leaving the region. The analyses of the subsequent cohorts suggest that whatever the determinants of longevity in the birth hotspot, they remain a factor over a period of at least 30 years.

The hotspots we have identified all seem more favorable to women. This is contrary to the situation for the Nicoya hotspot, where increased centenarian rates are seen only for men [5], and the hotspot in Sardinia, where the survival advantage is stronger for men than for women [1].

When looking for causes for the birth hotspot, we should note that this area historically has been a poor area with an agricultural economy dominated by large estates. The main island of Langeland experienced mass emigration for a period leading up to the birth of the cohort studied - 30% of the population emigrated in the period 1868-1909 as compared to a national average of 10% [13]. While blue zones are generally poor and rural areas [1], this may be a rare case of those left behind by emigration being the healthier group, an ‘unhealthy migrant-effect’. The landscape of Langeland is not markedly different from the rest of Denmark. Denmark is typically not considered a country with large genetic variation, but although the Danish population has historically been relatively mobile and there are no strong geographical barriers to mobility, there is still a tendency for surnames to cluster geographically, perhaps indicating lower mobility than might generally be the narrative [14].

The interpretation of the age 71 residence hotspots is less straightforward than that of the birth hotspot, due to the nature of migration. Place of residence at age 71 is an indicator of socioeconomic and health status, and thus a straightforward interpretation of the secondary hotspots as "the best places to grow old" is not possible. They can just as well be interpreted as "the places where the fittest live by age 71". The fact that the Northern Zealand hotspot was only beneficial for those born outside it might suggest that the effect is one of selective migration. Certainly, the Northern Zealand residential hotspot has markedly higher socioeconomic position by 1970 than the cohort in general and there could plausibly be selection factors into the Mid-Jutland hotspot as well.

Does a Danish longevity hotspot by place of birth exist? Our finding must be seen in the light of the limitations discussed: The relative increase in centenarian proportion is moderate as is the statistical significance level, and our results may be shaped by emigration or mortality before age 71. On the other hand, the hotspot observed persists for a period of at least 30 years, which makes it less likely to be merely a statistical fluke. In contrast to the primary residence hotspot, the place-of-birth hotspot is not immediately explained by socioeconomic factors. And so, even when scanning across a geographically and socially homogeneous country, centenarian hotspots can be found, although we can make no real guess as to the causes.

## METHODS

We constructed a cohort of men and women born in Denmark from 1906 to 1915 and still alive at the age of 71. For each person, we identified parish of birth and place of residence by age 71 plus date of death and emigration. The data source was the Population Registration System of Denmark, which allows individual follow-up for all persons resident in Denmark since 1968 [15]. Date of birth and place of residence are known for all persons in the registry, and all can be followed until death or emigration (whichever comes first). Parish of birth is recorded since 1977 for all persons alive and resident in Denmark. Information on later-life socioeconomic position and marital status was sourced from the 1970 census. We ended follow-up at Dec 1^st^ 2015.

The choice of cohort and of the reference age of 71 were pragmatic: We wanted to look at the most recent 10-year birth cohort that could have been recorded as centenarians by the time the study was begun, and as the oldest of this group had turned 71 by the time parish of birth was introduced as a register variable, we had to restrict to the subcohort surviving to this point. For simplicity, in the analyses of residence later in life, we chose to look at place of residence at age 71 rather than at some other age.

We searched for clusters of centenarians using a Kulldorff spatial scan [16] as implemented in the R package SpatialEpi [17]. For each parish, the number of centenarians and the expected number based on number of births by sex and year of birth were assigned to the geographical centroid of the parish. The method then constructed zones as circular areas containing up to a pre-specified proportion (we made the a priori choice to set this to 5%) of the total population. For each zone, a likelihood ratio test statistic was constructed assuming a Poisson distribution of the number of observed centenarians based on the expected number of centenarians. The zone most likely to be a hotspot was selected as the one maximizing the test statistic, and significance measures were computed by Monte Carlo simulation. Any other zones for which the test statistic was significant at the pre-specified significance level (set to 0.05) were flagged as secondary clusters.

Having detected a hotspot, we compared mortality and centenarian proportion in people born inside and outside the hotspot for men and women. Mortality was compared via hazard ratios computed by Cox regressions in which the outcome was death, and those who survived past the end of follow-up (Dec 31 2015) were censored. We also compared mortality and centenarian proportion according to whether the subjects were born and stayed in, had left, were born outside and had moved to, or were born and had stayed outside the cluster by age 71. We adjusted for birth year, socioeconomic position, marital status and homeownership. Missing values of covariates were set to the most common value for marital status, and to lowest for socioeconomic position.

In order to explore the extent to which the effect was stable over time, we compared cohort mortality for those born inside and outside the cluster over the period from 1978 to 2015 for the cohorts with birth years 1906-1915, 1916-1925 and 1926-1935. Five-year mortality rate ratios were computed by Poisson regression.

As a sensitivity analysis, since a large percentage of observations were coded with municipality rather than parish of birth, we performed cluster detection where the observations coded with municipality were weighted out across the parishes whose centroids were inside that municipality. The weights were proportional to the size of the birth cohort by year and sex in each parish.

We also performed cluster detection based not on the proportion of a birth cohort reaching age 100, but on the proportion of those residents in a region by age 71 who become centenarians.

Figure 1 and 2 show locations of the hotspots as well as smoothed centenarian proportions, where each parish is assigned the rate with numerator and denominator set to the respective averages for the nearest 20 parishes.

Analyses were done using R version 3.2.3 software.

## Supplementary Material

Supplementary Tables
