# Supplementary material for: Searching for longevity hotspots in Denmark
Source: Aging (Albany NY). 2018 Oct 13;10(10):2684–94. doi: 10.18632/aging.101579 (PMC6224260; doi:10.18632/aging.101579)
Supplement: Supplementary Tables [file aging-10-101579-s001.pdf]

SUPPLEMENTARY TABLES

Table S1. Parishes included in the birth cohort centenarian hotspot.

Cluster	Parishes included
Primary	Arninge, Avnede, Bjerreby, Branderslev, Bregninge, Bregninge, Brudager, Bøstrup, Dannemare, Drejø, Egense, Ellested, Fodslette, Frørup, Fuglsbølle, Gislev, Græshave, Gudbjerg, Gudme, Gurreby, Halsted, Hesselager, Horslunde, Hov, Humble, Hundstrup, Kappel, Kirkeby, Krarup, Kværndrup, Købelev, Landet, Langå, Lindelse, Longelse, Lunde, Magleby, Marstal, Nakskov, Nordlunde, Ollerup, Omø, Oure, Rise, Rudkøbing, Sandby, Sankt Jørgens, Sankt Nikolaj, Simmerbølle, Skovlænge, Skrøbelev, Skårup, Snøde, Stenstrup, Stoense, Stormark, Strynø, Svindinge, Sørup, Thurø, Tillitse, Tranderup, Tranekær, Tryggelev, Tullebølle, Tved, Ulbølle, Utterslev, Vejstrup, Vestenskov, Vester Skerninge, Vester Åby, Vesterborg, Vindeby, Vor Frue, Ærøskøbing, Øksendrup, Ørbæk, Øster Skerninge, Åstrup

Table S2. Parishes included in the primary and secondary residence-based centenarian hotspots.

Cluster	Parishes included
Primary	Bagsværd, Birkerød, Bistrup, Blovstrød, Buddinge, Christians, Dyssegård, Gammel Holte, Gentofte, Hellerup, Helleruplund, Hørsholm, Jægersborg, Karlebo, Kongens Lyngby, Lundtofte, Maglegårds, Ny Holte, Nærum, Ordrup, Rungsted, Skovshoved, Sorgenfri, Stengård, Søborggård, Søllerød, Tårbæk, Vangede, Vedbæk, Virum
Secondary	Arnborg, Assing, Aulum, Blåhøj, Bording, Brande, Brejning, Bryrup, Bølling, Egvad, Ejstrup, Engesvang, Faster, Filskov, Fredens, Funder, Gadbjerg, Give, Givskud, Gjellerup, Grene, Grindsted, Grove, Grædstrup, Hammer, Hanning, Havnstrup, Hedeager, Herborg, Herning, Hoven, Hvejsel, Ikast, Ilskov, Karup, Klovborg, Kragelund, Lindeballe, Linnerup, Nørre Snede, Nørre Vium, Nøvling, Oddum, Rind, Ringive, Sankt Johannes, Simmelkær, Sinding, Skarrild, Skjern, Snejbjerg, Studsgård, Sunds, Sædding, Sønder Borris, Sønder Felding, Sønder Omme, Them, Thorning, Thyregod, Timring, Tjørring, Tyrsting, Tørring, Vester, Videbæk, Vildbjerg, Vorgod, Vrads, Ølgod, Ørre, Øster Nykirke

Table S3. Mortality rate ratios comparing the birth cohort centenarian hotspot to the rest of Denmark, for the cohorts born 1906-15, 1916-25 and 1926-35.

	Birth year		
	**1906-1915**	**1916-1925**	**1926-1935**
	**Mortality rate ratio (95 % CI)**	**Mortality rate ratio (95 % CI)**	**Mortality rate ratio (95 % CI)**
**Population age 71, n**	362,064	367,269	339,258
**Born in hotspot, n**	12,472	12,365	10,559
**Age 71-75**	0.98 (0.94 - 1.02)	0.94 (0.90 - 0.98)	1.00 (0.95 - 1.05)
**Age 76-80**	0.98 (0.95 - 1.02)	0.91 (0.88 - 0.95)	0.98 (0.93 - 1.02)
**Age 81-85**	0.94 (0.90 - 0.97)	0.94 (0.91 - 0.98)	
**Age 86-90**	0.95 (0.91 - 0.98)	0.94 (0.90 - 0.98)	
**Age 90-95**	0.92 (0.87 - 0.97)		
**Age 96-100**	0.91 (0.84 - 0.99)		

Table S4. Hotspot by place of birth, when excluding and including observations coded with municipality of birth.

	Population	Centenarians, N (%)	Expected number of centenarians, N	Ratio, expected /observed
**Excluding observations with only municipality of birth recorded**				
**Birth hotspot**	12,472	222 (1.8)	161.9	1.37
**Rest of Denmark**	349,592	4,517 (1.3)	4,577.1	0.99
**Including observations with only municipality of birth recorded**				
**Birth hotspot**	13,590.7	240 (1.8)	180.5	1.33
**Rest of Denmark**	390,909.3	5,165 (1.3)	5,224.5	0.99
**Those with only municipality recorded**	42,436	666 (1.6)	580.9	1.15
